# Effects of Thermal Cycles on Mechanical Properties of RPECC: Static and Dynamic Splitting Tensile Performance

**DOI:** 10.3390/ma18050994

**Published:** 2025-02-24

**Authors:** Shaohua He, Huaqian Zhong, Zhiliang Chen, Huangwei Chen, Jincai Chen, Zhitao Yu

**Affiliations:** 1 School of Civil and Transportation Engineering, Guangdong University of Technology, Guangzhou 510006, China; hesh@gdut.edu.cn (S.H.); 2112209086@mail2.gdut.edu.cn (H.Z.); 2112309021@mail2.gdut.edu.cn (Z.C.); yuzt@gdut.edu.cn (Z.Y.); 2 Shanzhan Branch of Guangdong Road & Bridge Construction Development Co., Ltd., Meizhou 514000, China; 2112109007@mail2.gdut.edu.cn

**Keywords:** rubber powder, RPECC, thermal cycling, splitting tensile, thermal resistance

## Abstract

This paper examines the splitting tensile properties of rubberized polyethylene-engineered cementitious composites (RPECC) through static and dynamic experimental tests, highlighting the effects of thermal cycles, impact strain rates, and rubber powder substitution rates for fine aggregates. Damage patterns, ultimate tensile strength, time-dependent stress curves, dynamic failure strain, and the dynamic increase factor of the RPECC are presented. The microstructure of the material is analyzed using scanning electron microscopy and energy-dispersive X-ray spectroscopy. Experimental results reveal that incorporating rubber powders significantly enhances the deformability and ductility of RPECC in splitting tension. However, a high content of rubber powders, such as a substitution percentage of 30%, significantly reduces static and the dynamic ultimate tensile strength of the RPECC by 16.8% and 34.2%, respectively. Microstructural examinations indicate that thermal cycling weakens the internal adhesion between the rubber particles, polyethylene fibers, and the ECC matrix, resulting in the frequent withdrawal of fibers and the formation of calcium hydroxide, which diminishes the material tensile strength by up to 20.6% in static tests and 45.1% in dynamic tests. Despite these challenges, the RPECC with 20% rubber achieves a favorable balance between splitting the tensile properties and thermal resistance, even after undergoing 270 heat-cool cycles, suggesting its potential applicability in harsh environments.

## 1. Introduction

Engineered cementitious composites (ECC) are known for their exceptional mechanical properties, including a high tensile strength of up to 7 MPa and an ultimate tensile strain exceeding 3%, representing a 300- to 500-fold increase in ductility compared to traditional concrete [1,2]. Recently, ECC has gained popularity in infrastructure engineering, particularly in constructing concrete link slabs, which serve as a cost-effective alternative to traditional expansion joints in jointless bridges, such as steel rib joints, mat rubber expansion joints, and composite expansion joints. The advanced tensile capacity and multiple cracking characteristics of ECC link slabs enable them to accommodate significant axial deformation of the deck, effectively dissipate impact energy from traffic loads, and handle the complex stresses caused by thermal factors. To date, ECC link slabs have been implemented in several jointless bridge constructions, including a simply supported girder bridge in Michigan, USA [3], the Mihara Ohashi Bridge in Japan [4], and numerous newly established highway bridges in China [5].

Despite extensive research on the mechanical properties of ECC link slabs, recently established jointless bridges incorporating these slabs are still at high risk of crushing damage under typical service conditions, such as the excessive stresses caused by heavy traffic impact. A potential approach to improve the dynamic resilience of ECC link slabs is to incorporate waste rubber powders into a concrete matrix, resulting in a rubberized ECC (RPECC) [6,7]. Ye et al. [8] evaluated the impact of waste rubber on the dynamic properties of RPECC and found that increasing the rubber content from 0% to 10% significantly improved dynamic tensile ductility from 3.37% to 8.05%, accompanied by an 18.7% decrease in ultimate tensile strength. Similar findings were reported by Zhu et al. [9] and Su et al. [10], who demonstrated that the addition of waste rubber at levels of 5%, 10%, 20%, and 35% resulted in RPECC tensile ductility improvements of 41.2%, 17.9%, 71.6%, and 31.6%, respectively. Existing studies on the RPECC have demonstrated that adding rubber to ECC is efficacious in improving its tensile strength; however, the tensile properties of RPECC with varying percentages of rubber powders under static and dynamic loading remain unclear.

**Figure 1:**
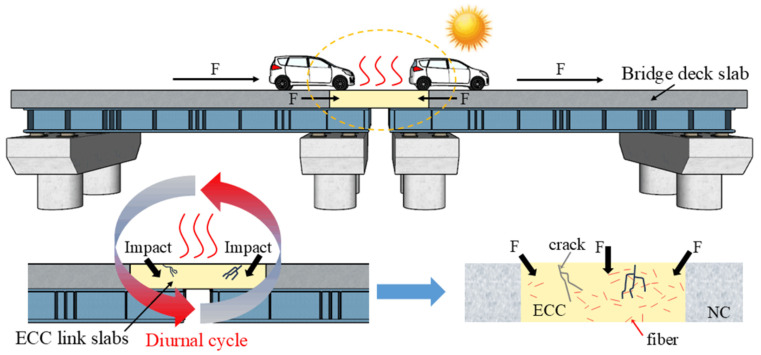
Damage mechanisms of ECC link slabs.

Although adding rubber to ECC is efficacious in improving its tensile properties at dynamic loading, recent studies indicate that the impact of temperature variation on RPECC mechanical properties cannot be overlooked, as is shown in Figure 1. Temperature stresses induced by the diurnal cycle can cause the continuous contraction and expansion of materials within RPECC. This repeated expansion and contraction may disrupt the microstructure of the RPECC, compromising its overall mechanical properties under external loading. Many researchers have begun to investigate the effect of temperature on the mechanical properties of RPECC. According to Mohammed et al. [11], the compressive strength of RPECC decreases with increasing temperature. This reduction is likely due to elevated temperatures altering the material’s microstructure and microchemical properties. Zhu et al. [9] observed that the dynamic properties of RPECC decrease at low temperatures, attributing this decrease to increased brittleness in the cementitious matrix caused by the freezing of pore water within the material. Similarly, the study conducted by Su et al. [12] reported a gradual decrease in the dynamic modulus of elasticity in RPECC with rising temperatures, which may be linked to pore expansion from the complete release of moisture and the formation of gaps due to the decomposition of rubber and fibers. The adverse effects of temperature on RPECC have been clearly documented by various researchers. However, most current studies on the mechanical properties of RPECC focus on tests conducted at constant temperatures, while research on its behavior under varying temperature conditions, particularly regarding its splitting tensile behavior during thermal cycling, remains limited.

This study aims to bridge this gap by investigating the splitting tensile properties of RPECC under thermal cycling conditions. It involves conducting both static and dynamic splitting tests focusing on failure modes, load–slip relationships, and dynamic increase factor (DIF) curves, utilizing advanced characterization techniques like scanning electron microscopy (SEM) and energy-dispersive X-ray spectroscopy (EDS) to uncover valuable insights into the material’s micro-damage mechanisms and stress transfer processes. This research provides a comprehensive assessment of RPECC’s tensile performance under thermally varying environments, with implications for its potential applications.

## 2. Experimental Program

### 2.1. Raw Materials and Mix Proportion

The raw materials used in this study included P⋅II 52.5 R Portland cement [13], fine-grained blast furnace slag powder [14], silica fume with a SiO_2_ content of over 70% (SF, SiO_2_ ≥ 70%) [15], quartz sand (QS) with a particle size range of 0.15–0.25 mm, powder rubber (PR), ultrahigh-molecular-weight polyethylene fiber (PE fiber), polycarboxylate high-range water reducer (HRWR), and water. Aligned with the principles of green recycling, the crumb rubber used in this study is sourced from waste rubber tires. The rubber particles are maintained at ambient temperatures, with an approximate granulometry range of 0.28–0.32 mm. The properties of ultrahigh-molecular-weight polyethylene fiber are shown in Table 1. Notably, the raw materials used in this study were sourced from Guangzhou, Guangdong Province.

Based on the experimental results reported by He et al. [16], the mass ratio of cement, fly ash, and blast furnace slag was 7.5:1:1.5. Polyethylene fibers and superplasticizers were included at 2% and 1% volume contents, respectively. The rubber powder was intended to replace sand in equal volume at substitution rates of 10%, 20%, and 30%. The specific material compositions are detailed in Table 2. In the specimen code, “C” means RPECC, “0, 90, 180, and 270” means that the number of thermal cycles is 0, 90, 180, and 270, respectively, and “10, 20, and 30” means that the rubber powder percentage content is 10%, 20%, and 30%.

### 2.2. Specimen Preparation

**Figure 2:**
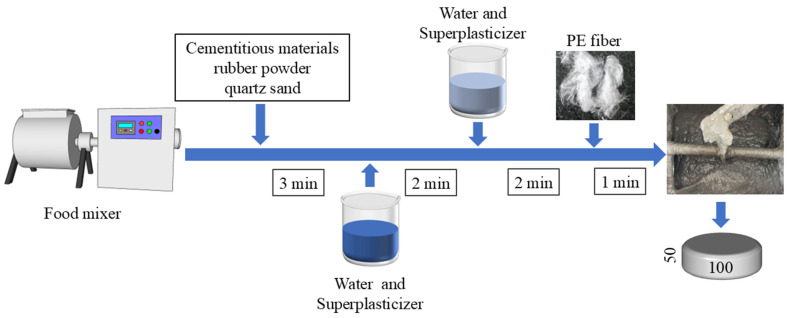
Preparation of RPECC specimens.

Cylindrical specimens (φ50 × 100 mm) were used for both static and dynamic tests of RPECC. The fabrication process is illustrated in Figure 2 and can be divided into five distinct steps: (i) the cementitious materials, rubber powder, and quartz sand are blended for three minutes to ensure homogeneity. (ii) A portion of the pre-mixed aqueous solution containing superplasticizer is introduced and mixed for an additional two minutes, then gradually pouring in the remainder of the solution. (iii) Continuously stirring the PE fibers within 1 min achieves uniform distribution. (iv) The thoroughly mixed RPECC is poured into a mold with dimensions of φ50 × 100 mm. After casting and consolidation, the specimens are covered with disposable plastic wrap to minimize rapid moisture loss and allow for ambient temperature curing for 24 h. (v) After the demolding phase, all specimens are placed in a controlled environment at 20 °C and 95% relative humidity for 28 days to facilitate the completion of the curing process.

### 2.3. Test Instruments and Procedure

#### 2.3.1. Thermal Cycle Program

**Figure 3:**
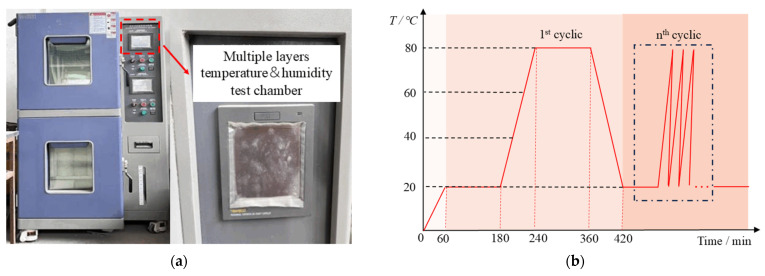
Instruments and procedures for thermal cycle: (**a**) test chamber; (**b**) schematic diagram of the thermal cycle.

The diurnal cycle refers to the natural alternation between day and night over time. The thermal cycling caused by this cycle can significantly impact the performance of ECC link slabs in tropical coastal regions [3,4,5]. To investigate the effects of thermal cycling on RPECC, this study utilized a multiple-layer temperature and humidity test chamber (Figure 3a) to simulate a tropical environment, following the thermal cycling pattern illustrated in Figure 3b. The experimental thermal cycling protocol was designed as follows: the temperature was varied between 20 °C and 80 °C, with the lower temperature (20 °C) maintained for 2 h and the higher temperature (80 °C) maintained for 2 h, following the procedures suggested in ASTM E831 [17]. The heating and cooling rates were controlled at 1.2 °C/min, with each phase lasting approximately 50 min, resulting in a total cycle duration of around 6 h. Additionally, the chamber humidity was maintained at 80% throughout the experiment to replicate the high-humidity conditions typical of tropical environments. One cycle (6 h) was used to simulate the temperature fluctuations of a summer day. Given that the summer season lasts approximately 90 days, the specimen was subjected to 90 thermal cycles to represent the seasonal temperature variations over the course of a year. Four loading scenarios were established: 0, 90, 180, and 270 cycles. The 270 cycles corresponded to three years of summer temperature cycling, with the design period aligned with the Chinese standard GB/T50082-2024 [18]. Notably, the multiple-layer temperature and humidity test chamber was sourced from Guangzhou, China.

#### 2.3.2. Static Loading Method

**Figure 4:**
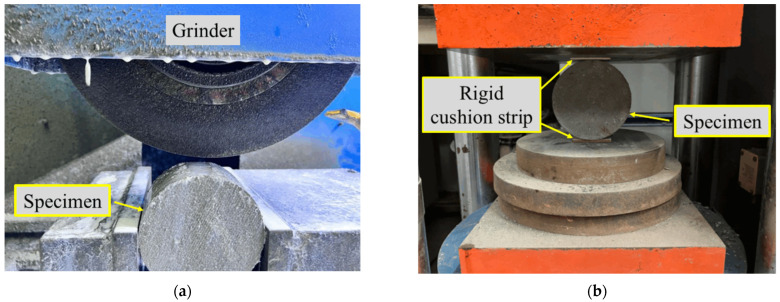
Grinding and static loading of RPECC: (**a**) specimen grinding; (**b**) static test.

A high-precision grinder (MY250), featuring a parallelism accuracy of 0.01/300 mm and a surface smoothness of Ra0.1 μm, was employed to grind the specimen prior to static loading. The grinding depth of the specimen was 0.76 mm, ensuring that the flatness of the specimen was maintained below 0.02 mm, as shown in Figure 4a. Notably, the high-precision grinder (MY250) and DYE-3000 pressure testing machine were sourced from Guangzhou, China.

In accordance with the Chinese standard GB/T 50081 [19], a static splitting tensile test was performed, utilizing the DYE-3000 pressure testing machine, as illustrated in Figure 4b. The specimen was securely positioned upright between the upper and lower rigid cushion strips and gradually loaded until it was firmly clamped in place. The initial load for the test was initiated at 3 kN, with the loading rate set to 0.1 kN/s, proceeding until the specimen split and ultimately failed. The splitting tensile strength was then derived using the prescribed formula:

(1) $$f_{\mathrm{ts}}=\frac{2F}{\pi A}$$

where f_ts_ is the splitting tensile strength (MPa); F is the specimen destructive load (N); A is the specimen splitting surface area (mm^2^).

#### 2.3.3. Dynamic Loading Method

**Figure 5:**
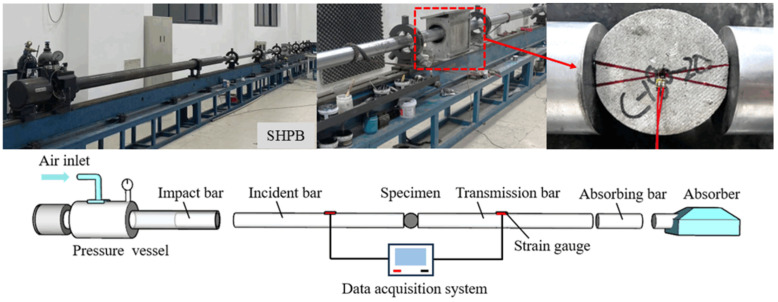
Dynamic splitting test with the Hopkinson pressure bar device.

Similar to static loading, the specimens were ground using a high-precision grinder (MY250) prior to dynamic loading. Dynamic splitting performance experiments were conducted using a split Hopkinson pressure bar (SHPB) device, which is made of high-strength steel materials for its foundational components, as shown in Figure 5. The split Hopkinson pressure bar (SHPB) device was sourced from Guangzhou, China. Semiconductor strain gauges were attached to both the incident and transmission rods to accurately measure key pulses: the incident pulse ε_i_(t) on the incident rod, the reflected pulse ε_r_(t) caused by the rod’s interaction with the specimen, and the transmitted pulse ε_t_(t) on the transmission rod. An additional strain gauge was attached to each specimen’s center to measure the failure strain, which was calculated by dividing the change in length by the original length of the specimen at the failure time. The formula for failure strain is:

(2) $$\epsilon_{f}=\frac{\Delta L}{L}$$

where ΔL is the change in length of the specimen and L is the original length of the specimen.

The loading procedure was based on the tests conducted by He et al. [16]; an output air pressure of 0.6–0.8 MPa ensured that the strain rate of the specimens remained within an appropriate range. The test begins by applying air pressure from the gun barrel to propel the impact rod into the incident rod. Upon impact, the strain gauges on the incident rod record the incident pulse, followed by the collision of the incident rod with the specimen and then with the transmission rod. The strain gauges on the transmission rod then record the transmission pulse ε_t_(t). Simultaneously, the remaining pulse reflects back toward the incident rod, traveling along the same path as the incident pulse ε_i_(t), and is recorded as the reflected pulse ε_r_(t).

The SHPB experiment is based on the theory of one-dimensional stress wave propagation in rods [20,21]. In dynamic splitting tests, the specimens used have central symmetry and parallel ends, following the principles of Brazilian disc splitting tensile tests [22], which allow crack initiation precisely from the center of the specimen. The tensile strength of the specimen material can be accurately determined using the following formula:

(3) $$\sigma_{t\;}=0.95\frac{2P}{\pi \mathrm{DL}}$$

where P is the applied load (N); D is the specimen diameter (mm); L is the specimen length (mm); and σ_t_ is tensile strength (MPa).

The applied load (P) to the specimen is calculated using the following equation [23].

(4) $$P\left(t\right)=\frac{E_{0}A}{2}(\varepsilon_{i}\left(t\right)+\varepsilon_{r}\left(t\right)+\varepsilon_{t}\left(t\right))$$

(5) $$\varepsilon_{i}\left(t\right)+\varepsilon_{r}\left(t\right)=\varepsilon_{t}\left(t\right)$$

where E_0_ and A denote the elastic modulus (MPa) and cross-sectional area (mm^2^) of the bars, respectively; ε_i_(t), ε_r_(t), and ε_t_(t) stand for the incident pulse, reflected pulse, and transmission pulse, respectively.

When combining Equations (4) and (5), the following Equation (6) to calculate the dynamic splitting tensile strength of the specimen is achieved:

(6) $$\sigma_{t}=0.95\frac{2P_{t,\max}}{\pi \mathrm{DL}}$$

where P_t,max_ is the maximum of P(t); σ_t_ is tensile strength (MPa).

The stress rate and strain rate are calculated using the following equation [24]:

(7) $$\sigma =\frac{f_{\mathrm{td}}}{\tau };\;\varepsilon =\frac{\sigma }{E}$$

where τ is the time interval from the initial position to the maximum value of the stress wave (s); σ is the stress rate (MPa/s); ε is the strain rate (s^−1^); E is the Young’s modulus of the specimen (MPa).

#### 2.3.4. Scanning Electron Microscopy

**Figure 6:**
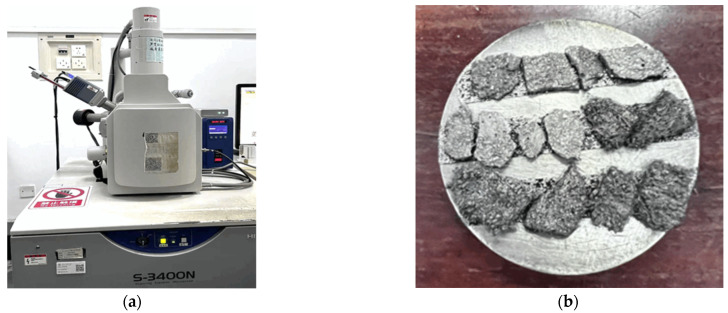
SEM device and sample preparation: (**a**) SEM; (**b**) prepared sample.

The microscope has a magnification range from 5 to 300,000 and a secondary electron image resolution of up to 3.0 nm, as shown in Figure 6a. The microstructure of RPECC under the static loading test and the dynamic loading test was examined using an S-3400N-II scanning electron microscope (SEM). Since the RPECC specimens were non-conductive, they were gold-coated using a vacuum deposition system prior to SEM analysis, as depicted in Figure 6b. The S-3400N-II scanning electron microscope (SEM) was sourced from Guangzhou, China.

## 3. Results and Discussion

### 3.1. Static Split Behavior

#### 3.1.1. Failure Pattern of Static Split Samples

**Figure 7:**
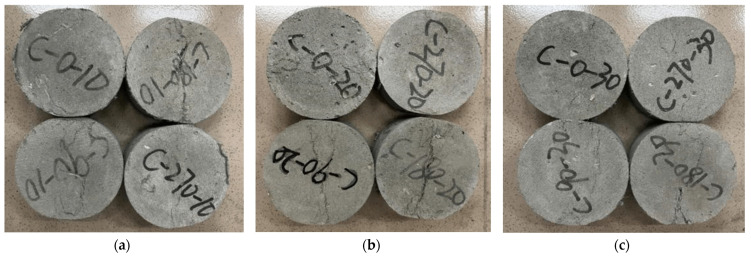
Static split failure mode of RPECC: (**a**) RPECC with 10% rubber content; (**b**) RPECC with 20% rubber content; (**c**) RPECC with 30% rubber content.

Figure 7 presents the failure patterns observed in static loading specimens. The RPECC exhibits a typical static damage pattern, maintaining an uninterrupted level of structural integrity. The damage cracking propagates consistently along the central axis, dividing the specimen into two symmetrical halves. The central crack is accompanied by a proliferation of microcracks, underscoring the distinctive strain-hardening attribute of ECC. This unique mechanism effectively redirects external loads through the reinforcing fibers to the uncracked matrix, enhancing its load-bearing capacity. Notably, the static damage patterns of ECC barely change with increasing rubber substitution. In contrast, specimens exposed to numerous thermal cycles exhibit different damage patterns, characterized by a marked reduction in microcracks. This phenomenon could be attributed to high-frequency thermal cycling, which promotes material discreteness within the specimens while diminishing their synergistic stress resilience, that is, the comprehensive ability of the material’s components to resist stress and absorb energy, ultimately changing the strain-hardening attribute of ECC.

#### 3.1.2. Static Splitting Tensile Properties

The results of the static splitting tensile test for RPECC are shown in Table 3. The static splitting tensile strength of RPECC containing 10% rubber decreased by 7.8% compared to ECC without rubber, as reported by Chen et al. [25]. This decline is attributed to the introduction of rubber powder, which impedes the hydration reaction between cement and water, resulting in excessive defects (pores, cracks, incompletely mixed aggregates, etc.) in the concrete. These defects serve as stress concentration points during loading, increasing the material’s susceptibility to fracture and leading to a marked reduction in tensile strength [26]. As the proportion of rubber powder doping increases, the static splitting tensile strength of RPECC decreases. For instance, specimens C-0-20 and C-0-30 exhibited significant reductions in split tensile strength, decreasing by 15.2% and 16.8%, respectively, compared to specimen C-0-10.

In contrast, after undergoing thermal cycles, RPECC’s static splitting tensile strength demonstrates a significant decrease. Specifically, RPECC with 10% rubber content, after undergoing 90, 180, and 270 thermal cycles, decreases its splitting strength by 13.9%, 19.7%, and 20.6%, respectively. This decline is attributed to the combined effects of thermal expansion and contraction, which increase the internal porosity of the sample and progressively deteriorate the interface transition zones between the different material phases within RPECC, resulting in compromised stress transfer and reduced split tensile strength.

**Figure 8:**
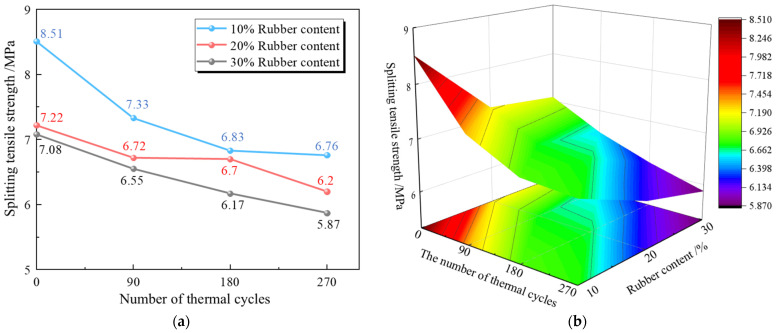
Static splitting test results: (**a**) splitting tensile strength curve; (**b**) splitting tensile strength distribution.

Figure 8a,b illustrates the static splitting tensile strength of RPECC after different numbers of thermal cycles. The results indicate that the adverse impact of thermal cycles on static splitting tensile strength was notably mitigated when the rubber content reached 20%. Specifically, the splitting strength of RPECC with 20% rubber decreased by only 6.9%, 7.2%, and 14.1% after 90, 180, and 270 thermal cycles, respectively. Meanwhile, RPECC with 30% rubber followed a similar trend but showed slightly more pronounced decreases of 5.1%, 12.8%, and 17.1%, respectively. In contrast, RPECC with 10% rubber experienced a more pronounced decline, with decreases of 13.9%, 19.7%, and 20.6%, respectively. Upon examining Figure 8b, it can be observed that when the rubber content in RPECC is at 20%, the material exhibits favorable thermal stability, characterized by a gradually decreasing trend rather than a steep decline. Although this experiment covered only a limited range of rubber content samples, it suggests that a rubber content of approximately 20% may optimize the thermal stability of RPECC. However, additional testing is required to determine the optimal rubber content that can maintain the heat resistance of RPECC.

### 3.2. Dynamic Split Behavior

#### 3.2.1. Failure Pattern of Dynamic Split Samples

**Figure 9:**
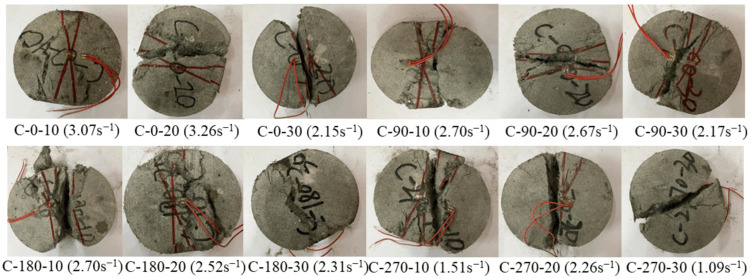
Typical dynamic split failure modes of RPECC.

Figure 9 displays the typical dynamic failure pattern of RPECC. Different from the static splitting failure pattern, the dynamic failure pattern can be divided into two types: (i) partial failure, where, due to the multi-seam cracking properties of ECC, a large number of penetration cracks appeared on the surface of the specimen, forming crushed triangular areas at its ends. These crushing triangles, which primarily appeared at the loaded end, were mainly connected to the central concrete. The specimens remained connected thanks to the fibers’ bridging effect, which is consistent with previous studies’ results [27,28]; (ii) complete failure, where impact loading causes cracks to propagate along the center of the specimen, resulting in a complete fracture down the middle, accompanied by a significant number of fragments. This behavior exhibited the characteristics of a brittle fracture.

The type of dynamic failure pattern is influenced by the rubber content. Due to the superior deformation capacity of rubber, ECC samples containing rubber exhibited more pronounced extrusion deformation when subjected to impact loads. In contrast, the rubber-free ECC displayed only penetrating cracks in the middle of the specimen [25]. As the rubber content increased, the specimens became more prone to complete failure, with a higher number of cracks and concrete fragments being formed. This is mainly due to the excess rubber powder, which exacerbates the initial flaws within the RPECC and facilitates crack propagation along these flaws under impact. This results in enhanced transverse deformation and more severe crushing. C-0-10 exhibited significant surface cracking, but the specimen did not undergo complete separation. In contrast, C-0-20 and C-0-30 were split entirely in half, due to the development of a central penetration crack. The bridging effect of the internal fibers within the specimen gradually diminishes as the number of thermal cycles increases. For instance, after 270 thermal cycles, specimen C-270-10 experienced a severe fracture and broke into smaller pieces compared to C-0-10. This suggests that thermal cycling damages the connections between the aggregate and the fiber in the ECC, making it more susceptible to fragmentation.

#### 3.2.2. Dynamic Splitting Tensile Properties

Table 4 summarizes the dynamic splitting tensile results of RPECC. One of the specimen sets, C-0-20, lacked data because its strain rate fell outside the expected range. Similar to the splitting behavior of conventional rubberized concrete [29], the splitting tensile strength increases significantly as the strain rate goes up, and a noticeable enhancement effect at higher strain rates was observed. For example, the dynamic splitting strength of C-0-10 was enhanced by 21.1% and 46.3% as the strain rate changed from 3.07 s^−1^ to 4.30 s^−1^ and then to 4.97 s^−1^, respectively. Similarly, C-0-30 experienced 14.4% and 34.6% increases with strain rate advancement from 2.15 s^−1^ to 2.89 s^−1^ and then to 3.58 s^−1^. This enhancement is due to the faster transmission of impact loads, which directs most of the impact energy along the primary crack to the high-strength rubber-cement matrix of RPECC.

Due to the excellent energy absorption capacity of rubber, RPECC demonstrates superior impact resistance under dynamic loading. The dynamic splitting tensile strength of RPECC with 10% rubber increased by 20.9% compared to rubber-free ECC [30]. However, as the rubber content increased, the dynamic splitting tensile strength decreased. For instance, the dynamic splitting strength of RPECC containing 20% rubber and RPECC containing 20% rubber decreased by 0.4–11.7% and 34.2–39.5%, respectively, compared to the RPECC containing 10% rubber. This reduction in strength occurs because excessive rubber inhibits the formation of a rigid core structure within the material, compromising its overall strength. In contrast, RPECC containing 20% rubber experienced a reduction in splitting strength by 23.6–34.4%, 31.0–37.9%, and 27.1–41.3% after 90, 180, and 270 thermal cycles, respectively. This can be explained by the thermal cycle diminishing the load-transfer efficiency of the fibers and deteriorating the bonding of different component materials, subsequently weakening the substrate strength.

**Figure 10:**
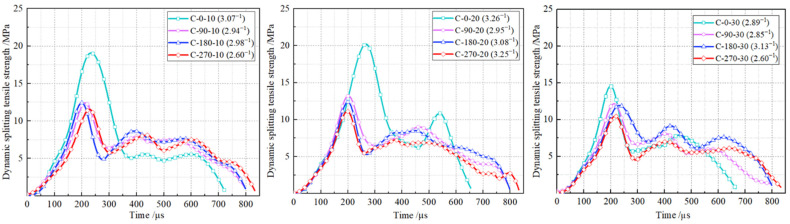
Dynamic splitting tensile stress–time curves of RPECC.

Figure 10 presents the time-dependent dynamic splitting tensile stress profiles for RPECC within the strain rate range of 2.60 s^−1^ to 3.30 s^−1^. The curves increase to a peak with loading time, typically reaching maximum stress at approximately 250 μs. This is followed by a multi-peak decline, which can be attributed to the bridging effect of the PE fibers at the cracked interface. Instead of sudden destruction, the specimens exhibited a gradual fracture, indicating the progressive degradation of the fibers and a transfer of energy to the uncracked matrix. Eventually, damage to the specimen occurs around 750 μs. It is evident that the time-dependent splitting tensile stress curves of RPECC after thermal cycles mostly follow the same trend as those observed without thermal cycling. In particular, the dynamic peaks decreased significantly after thermal cycling, which can be attributed to the weakening of the internal core structure of the RPECC due to the thermal cycling. As the duration of thermal cycling increased, the loading time frame remained consistently around 800 μs before termination. This indicates that the duration of thermal cycling had little effect on the ductility of RPECC.

#### 3.2.3. Dynamic Failure Strain Analysis

**Figure 11:**
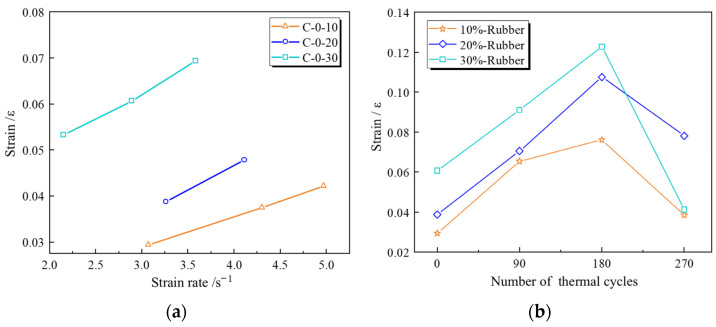
Dynamic failure strain analysis curves: (**a**) the effect of different strain rates; (**b**) the effect of different thermal cycles.

Figure 11 summarizes the failure strains of RPECC at different strain rates and thermal cycling conditions. The failure strain originates from the strain gauge at the center of the specimen. Figure 11a shows how RPECC’s failure strain increases with strain rate. Specifically, increasing the strain rate from 3.07 s^−1^ to 4.33 s^−1^ and 4.97 s^−1^ increased the failure strain of C-0-10 by 27.5% and 12.5%, respectively. Correspondingly, an increase in strain rate from 2.15 s^−1^ to 2.89 s^−1^ and 3.58 s^−1^ increased the failure strain of C-0-30 by 13.5% and 14.3%, respectively. The failure strains for C-0-30 were consistently higher than those for C-0-20 and C-0-10 across strain rates from 2.89 s^−1^ to 4.97 s^−1^, indicating the positive impact of rubber addition on material ductility and underscoring the crucial role of rubber content in shaping the material’s mechanical properties [31]. However, this improvement follows a complex relationship.

To understand the effect of different rubber contents on the mechanical behavior of RPECC under various thermal cycling conditions, a thorough data analysis was conducted within the strain rate range of 2.60 s^−1^ to 3.30 s^−1^, and the results are shown in Figure 11b. The failure strain of RPECC exhibited an initial increase, followed by a decrease in the number of thermal cycles. Specifically, for the sample with 10% rubber content, the failure strain increased from an initial value of 0.0294 to 0.0654 after 180 thermal cycles, representing an increase of 55.1%. It then continued to rise to 0.0761, marking a further increase of 14.1%. Similarly, for the specimen with 20% rubber content, the failure strain increased from 0.0388 to 0.0705 and then to 0.1076, corresponding to increases of 44.9% and 34.5%, respectively. For the 30% rubber content specimen, the failure strain increased from 0.0607 to 0.0910 and then to 0.1228, with increases of approximately 33.3% and 25.9%, respectively. These results indicate that the plastic deformation capacity of RPECC gradually improved with the number of thermal cycles during the first 180 cycles, demonstrating good thermal adaptability. As the number of thermal cycles increased to 270, the failure strain of the RPECC containing 10% rubber started to decrease sharply, dropping from 0.0706 to 0.0384, a 49.5% decrease, indicating the emergence of brittle characteristics. The RPECC samples with 20% or 30% rubber content also exhibited a declining trend. The 20% rubber content specimen decreased from 0.1076 to 0.0781, a reduction of around 27.4%, and the 30% rubber content specimen dropped from 0.1228 to 0.0412, a substantial decrease of approximately 66.5%. This trend highlights the detrimental effect of long-term thermal cycling on the brittle characteristics of RPECC material.

#### 3.2.4. Dynamic Increase Factor

**Figure 12:**
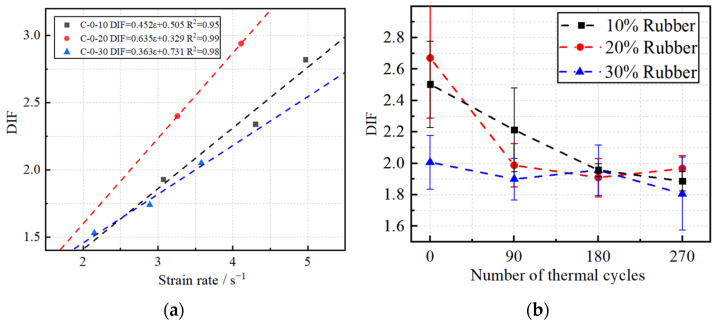
Relationships between RPECC and DIF: (**a**) DIF of RPECC at different strain rates; (**b**) DIF of RPECC at different thermal cycles.

The dynamic increase factor (DIF) is a critical parameter for evaluating the dynamic behavior of rubberized concrete [32]. It is defined as the ratio of dynamic to static splitting strength. Figure 12a illustrates the trend of the DIF for RPECC at different strain rates. As can be seen, an approximately linear increasing relationship existed between the DIF and strain rate. For example, the DIF of C-0-10 rose by approximately 21.2% and 20.5% as the strain rate increased from 3.07 s^−1^ to 4.30 s^−1^ and further to 4.97 s^−1^, respectively. The DIF of C-0-20 and C-0-30 also exhibited similar development trends with the strain rates. However, the slope for C-0-20 was higher than C-0-10 and C-0-30. This indicates that RPECC exhibits the most pronounced strain rate sensitivity with a 20% rubber content.

Figure 12b shows that the DIF values for all RPECC samples collectively decreased noticeably as the number of thermal cycles increased. A significant decrease in the DIF value was observed from the initial 0th cycle to the 90th cycle. The rate of decline slowed between the 90th and 270th cycles, indicating that early thermal cycles have a more significant impact on the splitting properties of RPECC. A possible explanation is that the internal densification of the material gradually decreases under the influence of thermal cycling, leading to a reduction in the strength of RPECC, but this detrimental effect begins to diminish with the increase in the number of thermal cycles. This also suggests the existence of a possible threshold for the number of thermal cycles within the temperature range of 20 °C to 80 °C, beyond which the effect of thermal cycling on RPECC is significantly diminished. It is worth mentioning that the DIF value for the 20% rubber content sample was slightly higher than those for the 10% and 30% rubber content samples after 270 thermal cycles. This suggests that RPECC exhibits optimal thermal cycling resistance when the rubber content is approximately 20%.

## 4. Microstructural Evolution

### 4.1. Micromorphological Analysis of RP-ECC

**Figure 13:**
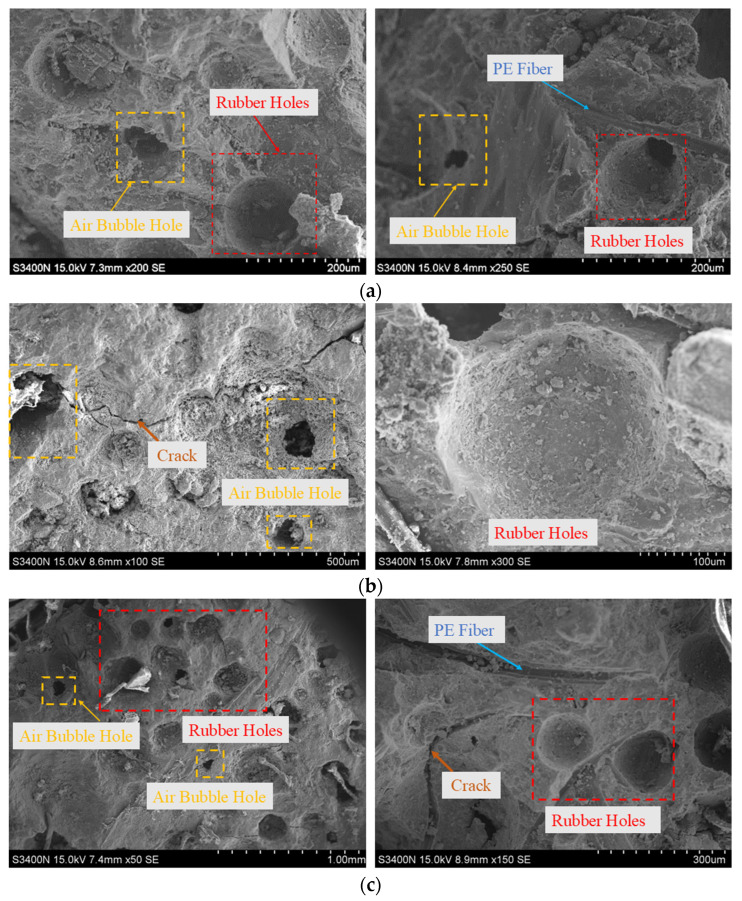
Internal structure of the RPECC matrix: (**a**) C-0-10; (**b**) C-0-20; (**c**) C-0-30.

Figure 13 shows the microscopic morphology of RPECC fragments containing 10% rubber, 20% rubber, and 30% rubber. It reveals a sparse presence of bubble defects and pores within the matrix. These defects result from incorporating rubber particles, which hinder chemical bonding between the materials and introduce air into the concrete mix. Some of these defects may also be casting residuals, due to insufficient vibration. It can be seen that increased rubber content leads to a proliferation of pores and additional defects, confirming that C-0-30 and C-0-20, with their higher rubber content, exhibit weaker splitting strength than C-0-10. This emphasizes the significant influence of rubber content on RPECC’s internal structure and mechanical properties.

**Figure 14:**
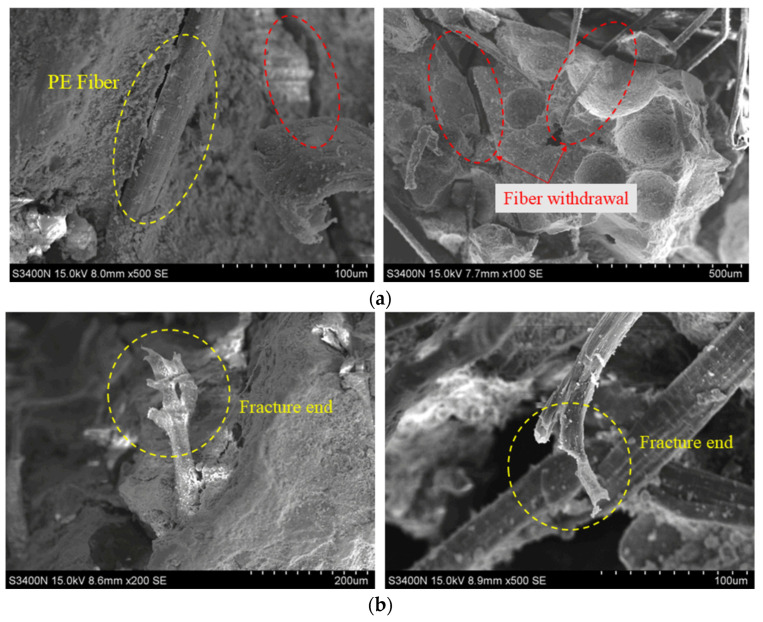
Failure pattern of PE fibers: (**a**) fiber withdrawal; (**b**) fiber fracture.

Figure 14 presents a detailed view of the microscopic structure of PE fibers in the matrix, highlighting two main types of damage: fiber withdrawal and fiber fracture. As shown in Figure 14a, the PE fibers appear relatively flat, but there are signs of detachment from the matrix, with visible traces of fiber extraction in the red circle. In contrast, Figure 14b shows a PE fiber under rapid loading, with one end tightening while the other remains embedded. This “pulling off” phenomenon demonstrates the fiber’s vital role as a load-transfer bridge during impact. It is important to note that rubber particles in the matrix only act as fillers and cannot form a dense, fiber-bridging structure through hydration reactions. As a result, fibers in the adhesive regions with rubber particles are particularly vulnerable to withdrawal or pulling-off mechanisms.

### 4.2. Microstructure Analyses of Energy-Dispersive X-Ray Spectroscopy

**Figure 15:**
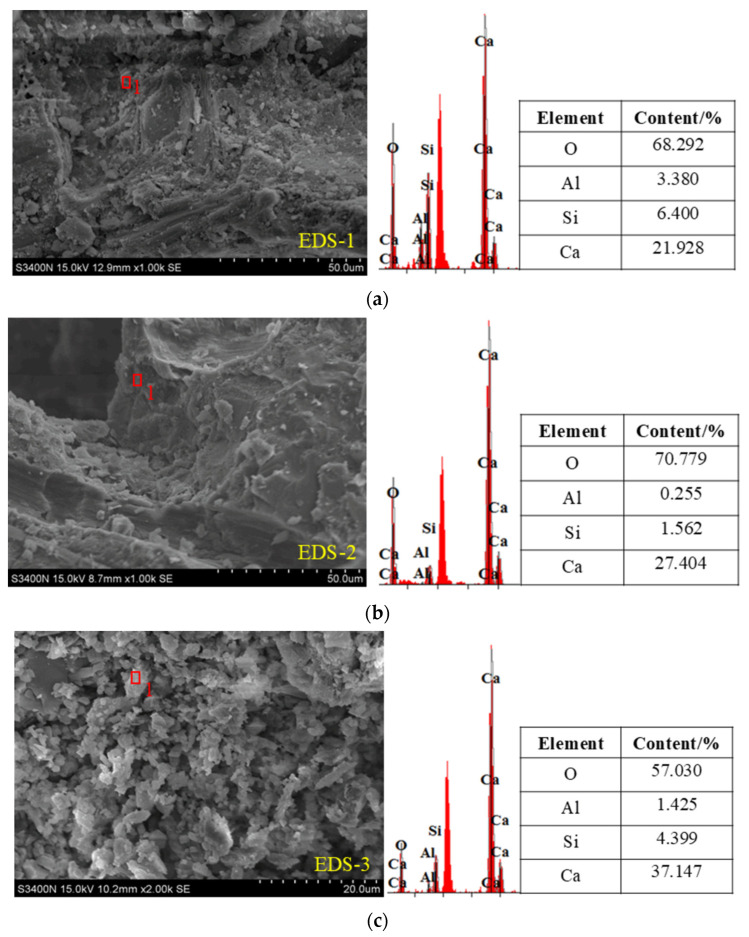

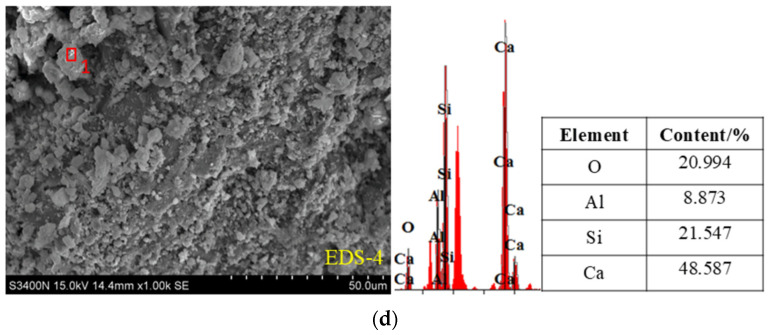
Microtopography images and EDS: (**a**) C-0-20; (**b**) C-90-20; (**c**) C-180-20; (**d**) C-270-20.

The image in Figure 15 shows the microscopic structure of the internal hydration products in RPECC containing 20% rubber, after undergoing different thermal cycles. Energy-dispersive X-ray spectroscopy (EDS) was used to analyze the RPECC component in detail. As the number of thermal cycles increased from 90 to 270, the internal structure of RPECC changed from a dense, layered formation with sporadic needle-like crystals to a distinct square and flocculent arrangement. This confirms that thermal cycling modifies the internal structure of ECC, accounting for the observed decrease in RPECC strength.

Examining the elemental compositions in Figure 15 reveals that the main components of these crystals are Si, O, Al, and Ca, indicating the presence of hydrated calcium silicate (C-S-H) gel, calcium hydroxide (CH), and Ettringite (AFt). AFt contributes to the early-age strength of cementitious materials, but, if not controlled, it may lead to volume expansion and cracking, affecting the long-term performance of RPECC. It is important to note that the similarity in calcium and oxygen contents suggests that the initial stages of thermal cycling promote the formation of dense C-S-H gels, which are beneficial for enhancing the strength and durability of the composite material. However, as the number of thermal cycles increases, these gels undergo further hydration reactions to produce lower-strength CH. This transformation creates weak areas within the internal structure, harming the structural strength and ultimately weakening the overall mechanical properties of RPECC [33]. Additionally, the presence of rubber particles may also affect RPECC’s hydration process and microstructural evolution. Rubber particles can act as micro-pores, altering the material’s internal porosity and moisture distribution. This, in turn, can impact the hydration reactions and the formation of hydration products. Therefore, the interplay between rubber content, thermal cycling, and hydration reactions is complex and requires further investigation to understand their full impact on the performance of RPECC.

## 5. Conclusions

This paper examines the static and dynamic splitting performance characteristics of rubberized polyethylene-engineered cementitious composites (RPECC), emphasizing failure modes, time-dependent splitting tensile stress curves, splitting strength, and the dynamic increase factor (DIF). The effects of impact strain rates, rubber content, and the number of thermal cycles on the splitting tensile properties of RPECC are explored. The principal conclusions drawn from this study are as follows.

1. The static and dynamic damage modes of RPECC differ significantly. Under dynamic loading, the damage is more severe, characterized by a central fracture and the formation of crushed triangular regions at both ends. In contrast, static loading results in the specimen breaking in half along the central axis.
2. Increasing the rubber powder content in RPECC decreases its split tensile strength, with reductions of 15.2% and 16.8% observed for static strength and 24.4% and 28.9% for dynamic strength for C-0-20 and C-0-30, respectively, compared to the C-0-10. However, due to rubber’s excellent deformability, higher rubber content enhances the ductility and deformability of RPECC, as evidenced by flatter dynamic splitting tensile stress–time curves and greater breaking strains.
3. Thermal cycling negatively affects the splitting tensile properties of RPECC. For instance, after 270 thermal cycles, the static splitting tensile strength of RPECC with 10% rubber content decreased by 20.6%, while those samples with 20% and 30% rubber content decreased by 14.1% and 17.1%, respectively, compared to specimens not subjected to thermal cycling. This indicates that thermal cycling increases the internal porosity of the sample and progressively deteriorates the interface transition zones between the different material phases within RPECC, which leads to the degradation of the splitting tensile properties of RPECC.
4. The dynamic increase factor (DIF) confirmed that RPECC with 20% rubber content exhibits excellent heat resistance, maintaining stable strength even after prolonged thermal cycling. This suggests that rubber, as a heat-resistant material, can effectively enhance the heat resistance of ECC, making its incorporation a promising approach.
5. SEM analysis of RPECC revealed that an increase in rubber content promotes the formation of porosity and additional defects, which, in turn, leads to a reduction in the strength of RPECC. EDS analysis of RPECC revealed that thermal cycling induces a hydration reaction within the material, producing an excess of calcium hydroxide (CH), contributing to a reduction in strength after cycling.

This study investigated the splitting tensile properties of RPECC under thermal cycling and examined its microstructure. The results confirmed that thermal cycling significantly affects the splitting and tensile properties of RPECC. However, RPECC with 20% rubber content exhibited improved heat resistance and deformation capacity. To effectively apply this approach, further research is needed to identify the optimal rubber content and to gain a deeper understanding of the compressive strength, tensile strength, and tensile strain resistance of ECC under thermal cycling. Future studies will focus on comprehensive mechanical testing to provide a more thorough and multidimensional understanding of RPECC’s mechanical properties for structural engineering applications.

## Figures and Tables

**Table 1:** Properties of PE fiber.

Length/mm	Diameter/um	Elongation at Break/%	Tensile Modulus/MPa	Tensile Strength/MPa	Density/(g/cm^3^)
18	24	2	116 × 10^3^	3000	0.97–0.98

**Table 2:** Mix proportions of RPECC.

Code	Cement/%	Fly Ash/%	Blast Furnace Slag/%	Water/%	Sand/%	Superplasticizer/%	PE Fiber/%	Thermal Cycles	Rubber Powder/%
C-0-10	43.68	5.82	8.74	14.56	26.21	1.0	2.0	0	10
C-0-20					23.29				20
C-0-30					20.38				30
C-90-10					26.21			90	10
C-90-20					23.29				20
C-90-30					20.38				30
C-180-10					26.21			180	10
C-180-20					23.29				20
C-180-30					20.38				30
C-270-10					26.21			270	10
C-270-20					23.29				20
C-270-30					20.38				30

**Table 3:** Static splitting tensile test results of RPECC.

Code		Splitting Tensile Strength/MPa	Average Value/MPa	Code		Splitting Tensile Strength/MPa	Average Value/MPa
C-0-10	a	8.75	8.51	C-180-10	a	7.15	6.83
	b	8.29			b	6.70	
	c	8.50			c	6.64	
C-0-20	a	6.82	7.22	C-180-20	a	6.59	6.70
	b	7.36			b	6.45	
	c	7.47			c	7.07	
C-0-30	a	7.24	7.08	C-180-30	a	6.30	6.17
	b	7.10			b	6.08	
	c	6.90			c	6.14	
C-90-10	a	7.53	7.33	C-270-10	a	6.89	6.76
	b	6.80			b	6.74	
	c	7.66			c	6.65	
C-90-20	a	6.66	6.72	C-270-20	a	6.33	6.20
	b	6.42			b	6.06	
	c	7.07			c	6.19	
C-90-30	a	6.12	6.55	C-270-30	a	6.10	5.87
	b	6.57			b	5.81	
	c	6.95			c	5.70	

**Table 4:** Dynamic splitting tensile test results.

Code	Strain Rate/s^−1^	Strength/MPa	Code	Strain Rate/s^−1^	Strength/MPa	Code	Strain Rate/s^−1^	Strength/MPa
C-0-10	3.07	16.42	C-0-20	3.26	17.35	C-0-30	2.15	10.80
	4.30	19.88		-	-		2.89	12.35
	4.97	24.03		4.11	21.21		3.58	14.54
C-90-10	2.70	14.56	C-90-20	2.67	13.25	C-90-30	2.17	11.47
	2.94	18.41		2.79	12.11		2.37	12.79
	6.44	15.65		2.95	13.91		2.85	13.09
C-180-10	2.70	13.34	C-180-20	2.52	11.82	C-180-30	2.31	11.13
	2.98	13.65		2.71	13.67		3.13	12.01
	3.60	13.14		3.08	13.18		3.23	13.05
C-270-10	1.51	12.71	C-270-20	2.26	12.65	C-270-30	1.09	9.06
	2.60	12.36		2.44	11.50		2.16	11.53
	2.64	13.19		3.25	12.42		2.60	11.25

## Data Availability

Some or all data, models, or codes that support the findings of this study are available from the corresponding author upon reasonable request. The data are not publicly available due to restrictions privacy.
